# Distal nerve transfers for ulnar nerve reinnervation and hand function restoration

**DOI:** 10.1016/j.bas.2026.106054

**Published:** 2026-04-15

**Authors:** Andrija Savić, Jovan Grujić, Aleksandar Djurdjević, Svetozar Stanković, Aleksa Mićić, Aleksandra Stojiljković, Branko Gaković, Milan Lepić, Nenad Novaković, Lukas Rasulić

**Affiliations:** a Faculty of Medicine, University of Belgrade, Belgrade, Serbia; b Clinic for Neurosurgery, University Clinical Centre of Serbia, Belgrade, Serbia; c Medical Faculty, Military Medical Academy, University of Defence, Belgrade, Serbia; d Clinic for Neurosurgery, Military Medical Academy, Belgrade, Serbia; e Clinic for Neurosurgery, University Clinical Centre of Kragujevac, Kragujevac, Serbia; f Clinic for Vascular Surgery, University Clinical Centre of Serbia, Belgrade, Serbia

**Keywords:** Ulnar nerve injury, Distal nerve transfer

## Abstract

**Introduction:**

Ulnar nerve injuries lead to severe intrinsic hand muscle dysfunction and major impairment of grip, pinch, and quality of life. Distal nerve transfers have been introduced to overcome the limitations of conventional repair and grafting by shortening the regeneration distance and improving reinnervation of intrinsic muscles.

**Research question:**

Can distal anterior interosseous nerve to deep motor branch of the ulnar nerve (AIN-to-UN-DMB) transfer restore intrinsic hand function and improve patient-reported quality of life in proximal ulnar nerve injuries?

**Material and methods:**

Twelve patients with proximal ulnar nerve injuries underwent distal AIN-to-UN-DMB transfer and were prospectively evaluated at 24 months postoperatively. Outcomes included intrinsic muscle strength graded by the BMRC scale, Egawa's sign, dynamometric assessment of grasp and pinch strength, and quality of life measured using the PNSQoL questionnaire.

**Results:**

At final follow-up, intrinsic muscle strength improved markedly: 27 muscles achieved M4 and 14 achieved M3 strength, compared with universal M0 preoperatively. Grasp strength increased from a mean of 42.3% to 82.5%, and pinch strength from 37.4% to 80.5% of the contralateral hand. Overall satisfactory functional recovery was achieved in 87.5% of patients. Mean PNSQoL score improved significantly from 47.7 preoperatively to 74.4 postoperatively (p < 0.001), with all patients reaching very good or excellent quality-of-life categories.

**Discussion and conclusion:**

Distal AIN-to-UN-DMB transfer enables meaningful restoration of intrinsic hand function, substantial gains in grasp and pinch strength, and significant improvement in quality of life, representing an effective reconstructive option for proximal ulnar nerve injuries.

## Highlights

• Distal AIN-to-ulnar motor transfer restores intrinsic hand muscle function.
• Significant improvement in grasp and pinch strength after surgery.
• Quality of life improves markedly following distal ulnar nerve reinnervation.
• Satisfactory functional recovery achieved in most patients at 2-year follow-up.

## 1 Introduction

The ulnar nerve is essential for intrinsic hand function, and its proximal injuries are among the most disabling lesions of the upper extremity. Such injuries often result in intrinsic muscle paralysis, claw hand deformity, sensory loss, and profound impairment of grip and pinch strength, severely limiting patients’ quality of life (Yoğun et al., 2025). Traditional reconstructive approaches, including direct repair and nerve grafting, often yield unsatisfactory results, due to the long regeneration distance and time-dependent degeneration of motor end plates, and up until recently, hand function recovery was considered impossible (Kim et al., 2003; Roganovic, 2004).

To overcome these limitations, distal nerve transfers have gained increasing importance. By redirecting expendable donor nerves to motor branches closer to target muscles, these procedures reduce regeneration distance, enhance reinnervation, and offer earlier functional recovery compared with conventional grafting (Fisher et al., 2024). Among them, the transfer of the terminal branch of the anterior interosseous nerve (AIN) to the deep motor branch of the ulnar nerve (UN-DMB) has become a distinguished strategy. First described by Mackinnon and colleagues, this technique can be performed in end-to-end or “supercharged” end-to-side fashion (Barbour et al., 2012). Clinical series and systematic reviews have reported encouraging results, with many patients achieving meaningful restoration of intrinsic motor function (Novak et al., 2002; Flores, 2015; Sallam et al., 2017). However, other studies have reported variable or limited benefits, highlighting ongoing debate and technical variability (Thorkildsen et al., 2025; Chambers et al., 2024).

Despite these controversies, distal AIN-to-UN-DMB transfer are increasingly incorporated into reconstructive strategies for high ulnar nerve injuries and severe cubital tunnel syndrome. Given the growing evidence but persisting variability in reported outcomes, more data is required to clarify the role of this approach.

We present a prospective series of 12 patients with proximal ulnar nerve injuries treated by distal AIN-to-UN-DMB transfer, with 2-year follow up for functional outcomes.

## 2 Methods

This series involved 12 patients, who underwent surgery at the Clinic for Neurosurgery, University Clinical Centre of Serbia in Belgrade, between 1st January 2016 and 31st December 2022 (Appendix 1). They were operated on because of the injures of the peripheral nerves of the upper extremities. The distal nerve transfer for the ulnar nerve was used and it included the transfer of the branch of the anterior interosseus nerve for the pronator quadratus muscle to the deep motor branch of the ulnar nerve (distal AIN-to-UN-DMB transfer)

The diagnostic methods used in the evaluation of the 12 patients included a detailed history, clinical and neurological examination, electrophysiological studies, and radiological studies (ultrasound, magnetic resonance).

Three weeks after the surgery, all patients underwent physical therapy for at least 12 weeks.

The final evaluation of the operative treatment was done two years after the surgery.

This study did not include the patients who, besides the injures of the peripheral nerves of the upper extremities, had associated injuries of the peripheral nerves of the lower extremities or associated injuries of the cranial nerves. The study also did not include the patients who did not give a written consent for the participation in the study, the patients who did not undergo a postoperative physical treatment in the duration of at least 12 weeks, and the patients who had not been coming to their follow-ups in the postoperative period of at least two years.

In the evaluation of hand function following the distal AIN-to-UN-DMB transfer, the following was analyzed: the strength of the adductor pollicis longus muscle - thumb adduction; the strength of the first dorsal interosseus muscle - index finger abduction; the presence of Egawa's sign in the middle finger; the strength of the lumbrical muscles, regarding ring finger flexion in the metacarpophalangeal joint, and extension in interphalangeal joints; the strength of the abductor digiti minimi muscle - fifth finger abduction; grasping strength; pinch strength; and the patients' quality of life.

The British Medical Research Council Scale (MRC) was used for the assessment of the strength of adductor pollicis, dorsal interossei muscles, thelumbrical and the abductor digiti minimi muscles. M0, M1, and M2 were considered unsatisfactory, while M3, M4, and M5 were designated as satisfactory useful functional recovery. In the group of functionally satisfactory recoveries, M5 is marked as excellent, M4 as very good, and M3 as good.

During the assessment, the patients were seated, the arm extended and leaning on the table, and in the position of protonation. During the assessment of the strength of the adductor pollicis muscle, the patients were asked to adduct the thumb against the examiner's resistance. During the assessment of the dorsal interossei muscles, the patients were asked to abduct the index finger against the examiner's resistance. During the assessment of the strength of the lumbrical muscles, the patients were asked to extend the interphalangeal joints against the examiner's resistance and while the examiner was holding the ring finger in hyperextension in the metacarpophalangeal joint. During the assessment of the strength of the abductor digiti minimi muscle, the patients were asked to abduct their little finger against examiner's resistance.

During the assessment of Egawa's sign, the patients were asked to flex their fingers in the interphalangeal and metacarpophalangeal joints, while the hand was leaning on the table and in the position of protonation, and then to both abduct and adduct their middle finger. The inability to perform this motion was marked as a positive Egawa's sign and vice versa.

The grasping and the pinch strength were examined dynamometrically by using ‘Jamar’ hand dynamometer (Lafayette Instrument, USA). The grasping or the pinch strength on the injured side was compared to the healthy side. The numerical values of the injured side were divided by the numerical values of the healthy side and multiplied by 100. The result is expressed as a percentage.

The analysis of the final treatment outcome in patients who underwent distal nerve transfer for ulnar nerve reconstruction, aimed at restoring intrinsic hand muscle function, was performed with particular emphasis on quality of life assessment using the Peripheral Nerve Surgery Quality of Life (PNSQoL) questionnaire—an original instrument developed at the Clinic for Neurosurgery, University Clinical Centre of Serbia (Appendix 2).

*All patients completed the Serbian version of the PNSQOL questionnairre.* The English version of the provided in this appendix represents a direct translation of the original Serbian instrument used in clinical practice and is included to facilitate understanding, interpretation, and reproducibility of the results for an international readership. The questionnaire consists of 16 items, each scored on a 6-point Likert scale (0–5), covering multiple domains including activities of daily living, social interaction, professional functioning, and subjective satisfaction. The instrument is designed to be simple, intuitive, and applicable across a wide range of peripheral nerve conditions.

The PNSQoL score ranges from 0 to 80, with lower values indicating poorer quality of life. For descriptive and comparative purposes, PNSQoL scores were categorized into five groups: poor (0–39), fair (40–49), good (50–59), very good (60–69), and excellent (70–80). Patients completed the PNSQoL questionnaire preoperatively and at 24 months postoperatively, and the results were subsequently analyzed.

### 2.1 Surgical procedure

Surgical procedure was performed using a standard volar approach to the forearm. During the transfer, the patient is supine, and the arm is in abduction. The pisiform bone and the flexor carpi ulnaris muscle serve as the reference points for the incision. Namely, the incision starts slightly laterally to the pisiform bone and extends up along the outer or the front border of the flexor carpi ulnaris muscle, and around the junction of the distal and the middle third of the forearm it turns upwards and laterally, and extends all the way to the level of the brachioradialis muscle and across the front side of the forearm.

Following the resection of the skin and the subcutaneous adipose tissue and its retraction, the forearm fascia is opened along the lateral edge of the flexor carpi ulnaris muscle, which is retracted medially, and the ulnar nerve and the artery are easily identified. It is necessary, through careful preparation, to separate the ulnar nerve from the ulnar artery in order not to damage it. It is then necessary to release the nerve proximally and distally, and to identify the sensory branch of the ulnar nerve for the dorsal side of the hand. One then accesses the ulnar artery, which can be separated from the flexor digitorum superficialis muscle, to which it tightly adheres. The ulnar artery gives off many branches positioned transversely towards the flexor digitorum superficialis muscle. At this point, these branches have to be cauterised and cut. This manoeuvre allows for the retraction of the flexor digitorum superficialis muscle laterally.

The flexor digitorum profundus muscle is then exposed and it is also retracted laterally. By doing this, the pronator quadratus muscle is exposed, and proximally from it the interosseous membrane, on which the terminal branch of the anterior interosseus nerve with the accompanying blood vessels lies. It should be noted that during this procedure the surgeon completely depends on their assistant, who by retracting the abovementioned muscles laterally, in a strong and continuous manner, creates the manoeuvring space for the surgeon. During the isolation of the terminal branch of the AIN for the pronator quadratus muscle, the surgeon needs to be very careful not to damage the accompanying blood vessel, which in such a small space can lead to profuse bleeding that is not easily controlled, and also avoid thermal damage to the nerve. It is also necessary to trace the terminal branch of the AIN for the pronator quadratus muscle as distally as possible and resect the fascia and the surface layer of the pronator quadratus muscle.

**Fig. 1:**
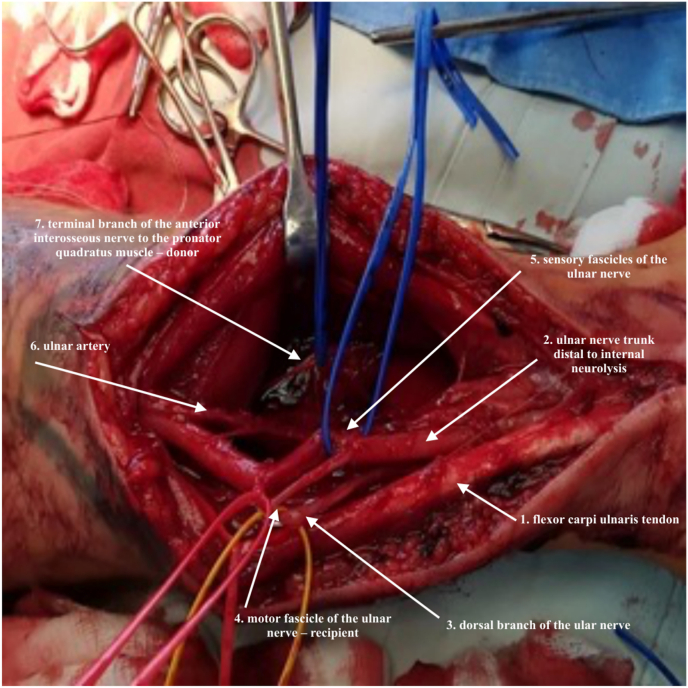
The terminal branch of the anterior interosseus nerve for the pronator quadratus muscle and the ulnar nerve following a neurolysis, at the level where its dorsal branch is isolated, and the motor and sensory fascicular groups are harvested.

**Fig. 2:**
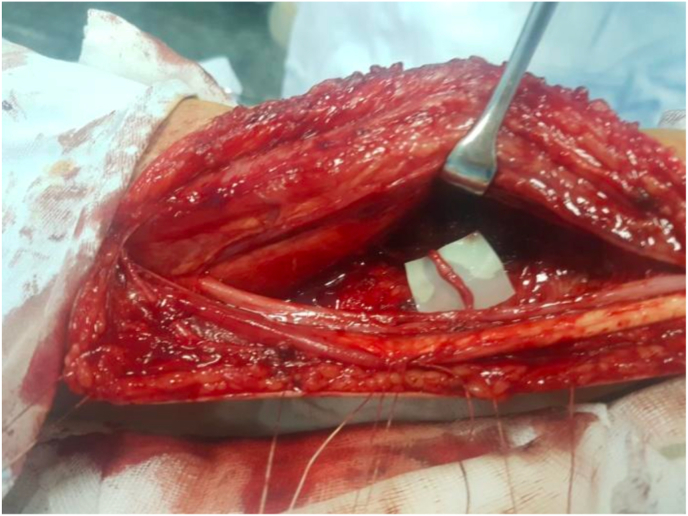
End-to-end coaptation between the terminal branch of the anterior interosseous nerve and the motor fascicle of the ulnar nerve.

The inner neurolysis of the ulnar nerve is performed next, immediately distally to the origin of the branch for the dorsal side of the hand. A thorough knowledge of the fascicular organisation of the ulnar nerve at this level is necessary. Namely, there are typically two fascicles – the upper one, which is larger and sensory, and the lower one, which is smaller and located between the upper sensory fascicle and the sensory branch of the ulnar nerve for the dorsal side of the hand (Fig. 1). This motor fascicle, the deep motor branch of the ulnar nerve the recipient, and ∼2 cm segment of it needs to be isolated. The terminal branch of the AIN for the pronator quadratus muscle is then cut as distally as possible and the motor fascicle of the ulnar nerve is transected as proximally as possible to allow for their direct coaptation (Fig. 2).

**Fig. 3:**
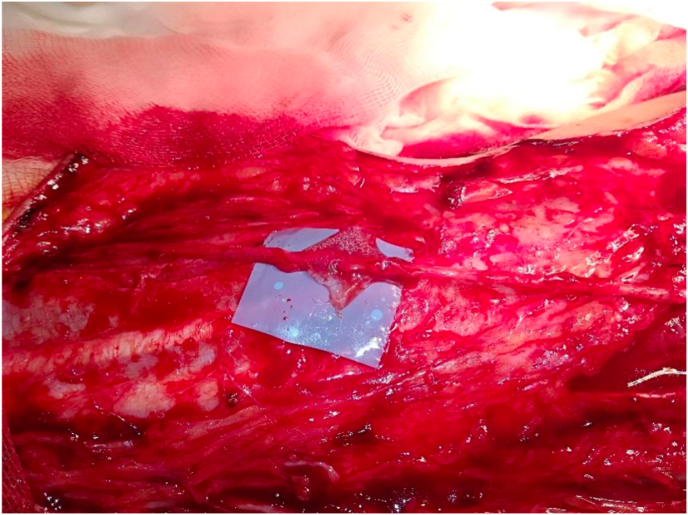
End-to-end coaptation between the lateral antebrachial cutaneous nerve and the sensory fascicle of the ulnar nerve.

Finally, at the level of the front lateral side of the forearm the lateral cutaneous nerve of the forearm is identified and released, followed by its distal section, mobilization medially, and a transfer to the sensory fascicle of the ulnar nerve for the palmar side of the hand, in order to secure protective sensation of the ulnar nerve (Fig. 3).

## 3 Results

In our series of 12 patients treated with distal anterior interosseous to ulnar motor nerve transfer, satisfactory functional recovery was achieved in the majority of cases. As shown in Table 1, intrinsic muscle strength improved markedly: while preoperatively 48 muscles were graded as M0, at final follow-up 27 muscles reached M4 strength and 14 achieved M3 strength. Although Egawa's sign remained positive in all patients, both grasping and pinch strength improved substantially. Average grasp strength increased from 42.3% preoperatively to 82.5% postoperatively, and pinch strength from 37.4% to 80.5%, reflecting significant functional gains. Overall satisfactory functional outcomes were achieved in 87.5% of cases regarding finger abduction and adduction, the flexion of 4th and 5th finger, and grasping and pinch strength.

**Table 1:** Final treatment outcome.

	Pre-op	Post-op
Intrinsic muscles' strength	48 M0	27 M4, 14 M3, 3 M2, 2 M1, 2 M0
Egawa's sign	12 +	12 +
Grasping strength	30%-55%, on average 42.3%	60%-95%, on average 82.5%
Pinch strength	23%-45%, on average 37.4%	55%-93%, on average 80.5%

Preoperatively, patients demonstrated predominantly impaired quality of life, with PNSQoL scores distributed across poor, fair, and good categories. Specifically, 2 patients (16.7%) were classified as having poor quality of life (0–39), 4 patients (33.3%) as fair (40–49), and 6 patients (50.0%) as good (50–59), while no patient achieved very good or excellent scores prior to surgery. Postoperatively, a marked shift toward higher quality-of-life categories was observed. One patient (8.3%) achieved a very good PNSQoL score (60–69), whereas the remaining 11 patients (91.7%) reached the excellent category (70–80). No patients remained in the poor, fair, or good categories after surgery (Table 2). The mean PNSQoL score increased significantly from 47.7 preoperatively to 74.4 postoperatively, representing a mean improvement of 26.8 points. This improvement was statistically significant on paired analysis using the Wilcoxon signed-rank test (p < 0.001), indicating a substantial and clinically meaningful enhancement in quality of life following surgical reconstruction.

**Table 2:** Distribution of patients according to PNSQoL categories before and after surgery.

PNSQoL category	Score range	Preoperative, n (%)	Postoperative, n (%)
*Poor*	*0–39*	*2 (16.7%)*	*0 (0%)*
*Fair*	*40–49*	*4 (33.3%)*	*0 (0%)*
*Good*	*50–59*	*6 (50.0%)*	*0 (0%)*
*Very good*	*60–69*	*0 (0%)*	*1 (8.3%)*
*Excellent*	*70–80*	*0 (0%)*	*11 (91.7%)*
***Total***		***12 (100%)***	***12 (100%)***

## 4 Discussion

This series of 12 patients with proximal ulnar nerve injuries treated with distal AIN-to-UN-DMB transfer demonstrated meaningful recovery of intrinsic muscle strength and improvement in grasp and pinch function.

Conventional reconstruction by direct repair or nerve grafting may restore flexor carpi ulnaris and the ulnar portion of the flexor digitorum profundus and provide protective sensation, but recovery of intrinsic muscle function remains disappointing in high lesions owing to the long regeneration distance and time-dependent degeneration of motor end plates (Kim et al., 2003; Roganovic, 2004). These limitations have motivated the increasing use of distal nerve transfers to reinnervate intrinsic muscles more quickly and physiologically (Fisher et al., 2024).

In this context, the distal AIN-to-UN-DMB transfer has emerged as a rational strategy, allowing dissection in uninjured planes, shortening the path to the motor end plates, and avoiding additional suture lines that waste regenerating axons. Our experience supports these advantages: most patients in our series achieved functionally satisfactory recovery of intrinsic strength together with marked gains in grasp and pinch, despite persistent Egawa's sign in all cases. These results are consistent with published series reporting meaningful restoration of intrinsic function after distal transfer and with comparative studies showing better motor outcomes than with grafting alone (Novak et al., 2002; Flores, 2015; Sallam et al., 2017).

Nevertheless, the literature is not unanimous. Comparative data indicate that the incremental benefit of adding an AIN transfer to primary repair may be modest in certain settings, with significant improvement sometimes confined to specific muscles such as the first dorsal interosseous (Yoğun et al., 2025). Similarly, clinical–neurophysiological assessments have shown that a demonstrable contribution of the transfer may be confirmed only in a subset of patients (Thorkildsen et al., 2025). These discrepancies likely reflect differences in patient selection, injury level and chronicity, timing of surgery, and technical execution. Our series provides a practical recommendation as younger patients and those treated within six months of injury recovered more readily, consistent with the general principle that earlier reinnervation before irreversible end-plate degeneration improves outcomes, while older delays diminish the probability of robust intrinsic recovery (Kim et al., 2003; Roganovic, 2004). Building on these observations, our findings allow for a more practical clinical interpretation. In our practice, distal AIN-to-UN-DMB transfer is primarily considered in patients with proximal ulnar nerve injuries presenting within six months after injury, particularly in the presence of severe intrinsic muscle weakness (M0–M1) and clinical signs of advanced denervation, such as clawing and a positive Egawa's sign. Patients presenting later or with more advanced muscle atrophy may still achieve partial functional improvement; however, outcomes are less predictable. Therefore, early referral and timely surgical intervention remain crucial to maximize the potential for intrinsic muscle reinnervation and functional recovery.

Technical variability across centers may also underlie heterogeneous results. A recent survey highlighted wide differences in whether a perineural window is created, whether partial fascicular injury is introduced, and where exactly the coaptation is placed on the ulnar motor component (Chambers et al., 2024). In our series, we performed a consistent end-to-end coaptation of the AIN branch to the deep motor branch, which may have contributed to the uniformity of results. The anatomical feasibility of this approach is supported by cadaveric data showing adequate length and axon counts in the AIN branch to pronator quadratus, with minimal donor morbidity because protonation is preserved via pronator teres (Sukegawa et al., 2014). These anatomical features help explain the reliability of distal transfer and its practical superiority to more proximal reconstructions.

Tendon transfers remain valuable as salvage procedures for correcting clawing and improving pinch in cases of absent or insufficient reinnervation; however, they are inherently non-physiologic, risk biomechanical imbalance and adhesions, and carry donor-site salvaging (Sammer et al., 2009). Our results suggest that when feasible, distal motor reinnervation should be prioritized, reserving tendon procedures for secondary augmentation in partial responders. Notably, even with satisfactory motor grades, residual signs such as persistent Egawa's may remain, indicating that distal transfer does not fully replicate native intrinsic coordination but still confers substantial functional benefit as reflected by the dynamometric improvements and the rise in patient-reported quality-of-life scores.

The diversity of injury mechanisms and associated lesions in our series - ranging from projectile and cut injuries to iatrogenic lesions and complex trauma - did not preclude favorable outcomes, which underscores the effectiveness of the distal approach when the recipient is close to the target and coaptation can be achieved without tension. Differences between lesions in continuity and nerve defects further illuminate the biology of recovery: better grasp restoration in continuity lesions likely derives from a greater number of regenerating axons that reach the ulnar-innervated flexors, whereas comparable pinch outcomes across groups reflect the dominant role of the distal transfer itself for intrinsic reinnervation, largely independent of spontaneous proximal regeneration.

Finally, our data align with broader trends showing growing clinical adoption of distal transfers for high ulnar lesions and severe cubital tunnel syndrome, despite ongoing debate (Fisher et al., 2024; Chambers et al., 2024). The balance of evidence favors distal AIN-to-UN-DMB transfer as a preferred reconstructive option in appropriately selected patients—especially younger individuals treated earlier—while acknowledging that outcomes vary and that adjunctive strategies (including staged tendon procedures) may be required in select cases.

It should be noted that the main limitation of this study is a small number of patients and the absence of a “blind review”, i.e. the preoperative evaluation, surgical treatment, and postoperative evaluation of the patients as well as the data collection, processing, analysis and the interpretation of the results were all done by the author of this study.

Future progress will depend on multicenter studies with harmonized indications, operative steps, and follow-up protocols to refine patient selection, quantify the specific contribution of the transfer versus native recovery, and optimize long-term functional results.

## 5 Conclusions

Distal nerve transfer for ulnar nerve reinnervation using anterior interosseous nerve to deep motor branch transfer enabled meaningful restoration of intrinsic hand function. In this series of 12 patients, overall satisfactory functional recovery was achieved in 87.5%, with marked improvements in intrinsic muscle strength and hand performance. Grasp strength increased from 42.3% to 82.5% and pinch strength from 37.4% to 80.5% compared with the contralateral hand. These functional gains translated into substantial improvements in patient-reported quality of life. Despite these encouraging outcomes, the evidence base in peripheral nerve surgery remains limited to single-center series, and meaningful advancement can only be achieved through coordinated multicentric collaboration and prospective study designs.

## Declaration of competing interest

The authors declare that they have no known competing financial interests or personal relationships that could have appeared to influence the work reported in this paper.
