# Supplementary material for: Distal nerve transfers for ulnar nerve reinnervation and hand function restoration
Source: Brain Spine. 2026 Apr 15;6:106054. doi: 10.1016/j.bas.2026.106054 (PMC13125893; doi:10.1016/j.bas.2026.106054)
Supplement: Multimedia component 1 [file mmc1.docx]

***Table 2***. Patients’ characteristics

| Number | Gender | Age | Type of injury | Nerve lesion type | Associated injuries | Emergency surgeries | Time between injury and surgery | Type of surgery | Results |
| --- | --- | --- | --- | --- | --- | --- | --- | --- | --- |
| 1 | M | 13 | SLF | IULc | FH, FU | HO, UO | 3 | ENU, PQ-DUB | I:M4 II:M4 III:+ IV:M4 V:M4 PROGS:47% POGS:90% PROPS:38% POPS:90% PRP:52 POP:78 |
| 2 | F | 21 | PI | RLcULd | / | / | 5 | ENR, GU, PQ-DUB | I:M4 II:M4 III:+ IV:M4 V:M4 PROGS:30% POGS:87% PROPS:23% POPS:80% PRP:30 POP:78 |
| 3 | M | 38 | C | IULd | BVL | BVR | 7 | PQ-DUB, III-SUB, D-ESM, III-ESM | I:M3 II:M3 III:+ IV:M3 V:M3 PROGS:55% POGS:82% PROPS:41% POPS:85% PRP:48 POP:71 |
| 4 | M | 36 | SLF | ICLBPU | / | / | 6 | ENU, PQ-DUB | I:M4 II:M4 III:+ IV:M3 V:M3 PROGS:35% POGS:80% PROPS:30% POPS:77% PRP:50 POP:75 |
| 5 | M | 37 | C | IULd | / | / | 6 | GU, PQ-DUB, PB-SUB | I:M4 II:M3 III:+ IV:M3 V:M3 PROGS:52% POGS:87% PROPS:37% POPS:83% PRP:54 POP:73 |
| 6 | M | 40 | HF | RLcULc | FF | FO | 7 | ENR, ENU, PQ-DUB | I:M4 II:M3 III:+ IV:M3 V:M4 PROGS:33% POGS-78% PROPS:40% POPS:75% PRP:43 POP:70 |
| 7 | M | 29 | C | IULd | / | / | 4 | GU, PQ-DUB | I:M4 II:M4 III:+ IV:M3 V:M4 PROGS:43% POGS:89% PROPS:35% POPS:86% PRP:51 POP:77 |
| 8 | M | 67 | C | IULd | BVL | BVR | 9 | PQ-DUB | I:M3 II:M2 III:+ IV:M1 V:M2 PROGS:49% POGS:69% PROPS:36% POPS:68% PRP:57 POP:71 |
| 9 | M | 28 | IA | IULd | / | / | 3 | PQ-DUB | I:M4 II:M4 III:+ IV:M4 V:M4 PROGS:43% POGS:88% PROPS:45% POPS:85% PRP:45 POP:79 |
| 10 | M | 58 | C | IULd | / | / | 7 | PQ-DUB | I:M2 II:M0 III:+ IV:M0 V:M1 PROGS:50% POGS:60% PROPS:40% POPS:55% PRP:55 POP:63 |
| 11 | M | 20 | C | IULd | / | / | 3 | GU, PQ-DUB | I:M4 II:M4 III:+ IV:M4 V:M4 PROGS:40% POGS:95% PROPS:44% POPS:93% PRP:48 POP:80 |
| 12 | M | 18 | TAD | ICLBPUR | MBF, MF, CC, CL | CS | 7 | TD-TGR, FCR-IP, FDS-ECRB, PQ-DBU, LCN-SUB | I:M4 II:M4 III:+ IV:M4 V:M3 PROGS:31% POGS:85% PROPS:40% POPS:89% PRP:39 POP:78 |

Abbreviations: M-male, F-female, SLF-same level fall, PI-projectile injury, C-cut, HF-fall from height, IA-iatrogenic injury, TAD- traffic accident with the driver in the car, IULc- the isolated lesion of the ulnar nerve in continuity in the upper arm, RLcULd- the lesion of the radial nerve in continuity and the lesion of the ulnar nerve in discontinuity at the level of the upper arm, IULd- the isolated lesion of the ulnar nerve in discontinuity in the upper arm, ICLBPU- infraclavicular lesion of the brachial plexus with a predominant affection of the ulnar nerve in continuity, RLcULc- the lesion of the radial and the ulnar nerve, both in continuity, ICLBPUR- infraclavicular lesion of the brachial plexus with a predominant affection of the ulnar nerve and the radial nerve both of which are in continuity, FH- humerus fracture, FU- ulnar fracture, BVL- blood vessel lesion, FF- femur fracture, MBF- metatarsal bone fracture, MF- mandible fracture, CC- cerebral contusion, CL- colon lesion, HO-humerus osteosynthesis, UO- ulna osteosynthesis, BVR- blood vessel reconstruction, FO- femur osteosynthesis, CS- colon suture, ENU- external neurolysis of the ulnar nerve, PQ-DUB – the transfer of the terminal branch of the anterior interosseus nerve for the pronator quadratus muscle to the deep motor branch of the ulnar nerve, ENR- external neurolysis of the radial nerve, GU- the graft of the ulnar nerve, III-SUB- the transfer of the sensory fascicle of the median nerve for 3^rd^ interdigital space to the palmar sensory branch of the ulnar nerve, D-ESM- he transfer of the dorsal sensory branch of the ulnar nerve for the median nerve “end to side”, III-ESM- the transfer of the remaining distal division of the sensory fascicle of the median nerve for the median nerve “end to side”, PB-SUB- the transfer of the palmar sensory branch of the median nerve to the palmar sensory branch of the ulnar nerve, TD-TGR- the transfer of the branch of the thoracodorsal nerve to the branch of the radial nerve for the long head of the triceps muscle, FCSR-IP- the transfer of the branch of the median muscle for the flexor carpi radialis muscle to the posterior interosseus nerve, FDS-ECRB- transfer of the branch of the median nerve for the flexor digitorum superficialis muscle to the branch of the radial nerve for the extensor carpi radialis brevis muscle, LCN-SUB- the transfer of the lateral cutaneous nerve of the forearm to the palmar sensory branch of the ulnar nerve, I- thumb adduction strength, II- index finger abduction strength, III- Egawa’s sign, IV- the strength of the medial lumbrical muscle at the level of the ring finger, V- little finger abduction strength, PROGS- preoperative grasping strength compared to the healthy side, POGS- postoperative grasping strength compared to the healthy side, PROPS-preoperative pinch strength compared to the healthy side, POPS- postoperative pinch strength compared to the healthy side, PRP – preoperative Peripheral Nerve Surgery Quality of Life (PNSQOL) questionnaire score, POP – postoperative Peripheral Nerve Surgery Quality of Life (PNSQOL) questionnaire score, M1,M2,M3,M4,M5- motion strength following the BMRC scale
