# Supplementary material for: Distal nerve transfers for ulnar nerve reinnervation and hand function restoration
Source: Brain Spine. 2026 Apr 15;6:106054. doi: 10.1016/j.bas.2026.106054 (PMC13125893; doi:10.1016/j.bas.2026.106054)
Supplement: Multimedia component 2 [file mmc2.docx]

**Appendix 2**

PNSQOL (Peripheral Nerve Surgery Quality of Life) questionnaire is an original questionnaire designed by the authors (Clinic for Neurosurgery, University Clinical Centre of Serbia) to improve quality of life (QOL) assessment in patients undergoing upper and/or lower extremity peripheral nerve surgery (PNS). The questionnaire provides a structured evaluation of a patient's ability to perform daily activities, social interactions, professional engagement, and overall satisfaction with their condition before and/or after surgery. Questions 13, 14, and 15 explicitly differentiate between a patient's state before or after surgery. The score represents a continuous variable, ranging from 0-80, with higher scores indicating better QOL. All answers must be provided to be valid. For descriptive and comparative analyses, PNSQoL scores were further categorized into five groups: poor (0–39), fair (40–49), good (50–59), very good (60–69), and excellent (70–80).The patients in this study completed the PNSQOL questionnaire translated to the Serbian language (Serbian version).

**PNSQOL questionnaire** (English version)

1. **How would you rate your ability to independently maintain personal hygiene (washing your face, brushing your teeth, showering, combing, drying your hair)?**

0- unable

1- able with extreme difficulty

2- able with great difficulty

3- able with moderate difficulty

4- able with difficulty

5- able without difficulty

1. **How would you rate your ability to dress yourself?**

0- unable

1- able with extreme difficulty

2- able with great difficulty

3- able with moderate difficulty

4- able with difficulty

5- able without difficulty

1. **How would you rate your ability to independently pick up a glass from a table and drink the content?**

0- unable

1- able with extreme difficulty

2- able with great difficulty

3- able with moderate difficulty

4- able with difficulty

5- able without difficulty

1. **How would you rate your ability to independently eat food someone else prepared and served on the table (use of cutlery)?**

0- unable

1- able with extreme difficulty

2- able with great difficulty

3- able with moderate difficulty

4- able with difficulty

5- able without difficulty

1. **How would you rate your ability to independently do housework (making the bed, folding clothes, cooking, vacuuming, throwing the trash away, gardening)?**

0- unable

1- able with extreme difficulty

2- able with great difficulty

3- able with moderate difficulty

4- able with difficulty

5- able without difficulty

1. **How would you rate your ability to go grocery shopping on your own?**

0- unable

1- able with extreme difficulty

2- able with great difficulty

3- able with moderate difficulty

4- able with difficulty

5- able without difficulty

1. **How would you rate your ability to participate in recreational activities (walking, running, swimming, playing football, playing basketball)?**

0- unable

1- able with extreme difficulty

2- able with great difficulty

3- able with moderate difficulty

4- able with difficulty

5- able without difficulty

1. **How would you rate your ability to continue with your work-related activities (going to school, going to university, going to work)?**

0- unable

1- able with extreme difficulty

2- able with great difficulty

3- able with moderate difficulty

4- able with difficulty

5- able without difficulty

1. **Do you have sleep problems because of the current condition of your extremity?**

0- Everyday

1- Almost every day

2- Often

3- Sometimes

4- Rarely

5- Never

1. **In the past month, have you experienced people from your surroundings feeling pity for you because of the current condition of your extremity?**

0- Everyday

1- Almost every day

2- Often

3- Sometimes

4- Rarely

5- Never

1. **In the past month, have you experienced humiliation or discrimination because of the current condition of your extremity?**

0- Everyday

1- Almost every day

2- Often

3- Sometimes

4- Rarely

5- Never

1. **In the past month, has your social life been influenced by the current condition of your extremity (relationship with your family, friends, colleagues)?**

0- Everyday

1- Almost everyday

2- Often

3- Sometimes

4- Rarely

5- Never

1. **In the past month, has the current condition of your extremity limited your everyday activities (taking care of yourself, taking care of your family, taking care of your household, going to school, going to university, going to work, doing work-related tasks)?**

0- Everyday

1- Almost every day

2- Often

3- Sometimes

4- Rarely

5- Never

1. **Are you satisfied with the current condition of your extremity compared to its condition before the symptom symptom/injury/disorder/disease onset or its condition before the surgery?**

0- Very unsatisfied

1- Moderately unsatisfied

2- Slightly unsatisfied

3- Slightly satisfied

4- Moderately satisfied

5- Very satisfied

1. **Are you satisfied with your current social life compared to your social life before the symptom/injury/disorder/disease onset or before the surgery?**

0- Very unsatisfied

1- Moderately unsatisfied

2- Slightly unsatisfied

3- Slightly satisfied

4- Moderately satisfied

5- Very satisfied

1. **Are you satisfied with your current professional life compared to your professional life before the symptom/injury/disorder/disease or before the surgery?**

0- Very unsatisfied

1- Moderately unsatisfied

2- Slightly unsatisfied

3- Slightly satisfied

4- Moderately satisfied

5- Very satisfied

**PNSQOL questionnaire** (Serbian version)

1. **Kako biste ocenili svoju sposobnost da samostalno održavate ličnu higijenu (umivanje, pranje zuba, tuširanje, češljanje, feniranje)?**
2. Nesposoban/na
3. Sposoban/na sa veoma velikim teškoćama
4. Sposoban/na sa velikim teškoćama
5. Sposoban/na a sa umerenim teškoćama
6. Sposoban/na sa lakim teškoćama
7. Sposoban/na bez teškoća
8. **Kako biste ocenili svoju sposobnost da se samostalno obučete?**
9. Nesposoban/na
10. Sposoban/na sa veoma velikim teškoćama
11. Sposoban/na sa velikim teškoćama
12. Sposoban/na sa umerenim teškoćama
13. Sposoban/na sa lakim teškoćama
14. Sposoban/na bez teškoća
15. **Kako biste ocenili svoju sposobnost da samostalno uzmete, sa stola, čašu vode i popijete je?**
16. Nesposoban/na
17. Sposoban/na sa veoma velikim teškoćama
18. Sposoban/na sa velikim teškoćama
19. Sposoban/na sa umerenim teškoćama
20. Sposoban/na sa lakim teškoćama
21. Sposoban/na bez teškoća
22. **Kako biste ocenili svoju sposobnost da samostalno jedete hranu koju vam je neko drugi pripremio i postavio na sto (korišćenje kašike, viljuške i noža)?**
23. Nesposoban
24. Sposoban/na sa veoma velikim teškoćama
25. Sposoban/na sa velikim teškoćama
26. Sposoban/na sa umerenim teškoćama
27. Sposoban/na sa lakim teškoćama
28. Sposoban/na bez teškoća
29. **Kako biste ocenili svoju sposobnost da samostalno obavljate kućne poslove (raspremanje kreveta, slaganje odeće, kuvanje, usisavanje, bacanje smeća, sredjivanje dvorišta)?**
30. Nesposoban
31. Sposoban/na sa veoma velikim teškoćama
32. Sposoban/na sa velikim teškoćama
33. Sposoban/na sa umerenim teškoćama
34. Sposoban/na sa lakim teškoćama
35. Sposoban/na bez teškoća
36. **Kako biste ocenili svoju sposobnost samostalnog odlaska u prodavnicu radi kupovine namirnica za kuću?**
37. Nesposoban/na
38. Sposoban/na sa veoma velikim teškoćama
39. Sposoban/na sa velikim teškoćama
40. Sposoban/na sa umerenim teškoćama
41. Sposoban/na sa lakim teškoćama
42. Sposoban/na bez teškoća
43. **Kako biste ocenili svoju sposobnost učestvovanja u rekreativnim aktivnostima (šetnja, trčanje, plivanje, fudbal, basket)?**
44. Nesposoban/na
45. Sposoban/na sa veoma velikim teškoćama
46. Sposoban/na sa velikim teškoćama
47. Sposoban/na sa umerenim teškoćama
48. Sposoban/na sa lakim teškoćama
49. Sposoban/na bez teškoća
50. **Kako biste ocenili svoju sposobnost da nastavite sa svojim radnim aktivnostima (učenje škole, studiranje, rad na svom radnom mestu)?**
51. Nesposoban/na
52. Sposoban/na sa veoma velikim teškoćama
53. Sposoban/na sa velikim teškoćama
54. Sposoban/na sa umerenim teškoćama
55. Sposoban/na sa lakim teškoćama
56. Sposoban/na bez teškoća
57. **Da li zbog trenutnog stanja vašeg ekstremiteta imate probleme sa spavanjem?**
58. Svakodnevno
59. Skoro svakodnevno
60. Često
61. Ponekad
62. Retko
63. Nikad
64. **Da li ste zbog trenutnog stanja vašeg ekstremiteta osetili sažaljenje ljudi iz vaše okoline u poslednjih mesec dana?**
65. Svakodnevno
66. Skoro svakodnevno
67. Često
68. Ponekad
69. Retko
70. Nikad
71. **Da li ste zbog trenutnog stanja vašeg ekstremiteta doživeli poniženje ili diskriminaciju u poslednjih mesec dana?**
72. Svakodnevno
73. Skoro svakodnevno
74. Često
75. Ponekad
76. Retko
77. Nikad
78. **Da li je stanje vašeg ekstremiteta u poslednjih mesec dana uticalo na vaš socijalni život (odnos sa porodicom, prijateljima, kolegama)?**
79. Svakodnevno
80. Skoro svakodnevno
81. Često
82. Ponekad
83. Retko
84. Nikad
85. **Da li vas je stanje ekstremiteta u poslednjih mesec dana ograničavalo u izvođenju svakodnevnih radnih aktivnosti (briga o sebi, briga o porodici, briga o kući, odlazak u školu, odlazak na fakultet, odlazak na posao, izvođenje predviđenih radnih zadataka)?**
86. Svakodnevno
87. Skoro svakodnevno
88. Često
89. Ponekad
90. Retko
91. Nikad
92. **Da li ste zadovoljni trenutnim stanjem vašeg ekstremiteta u odnosu na stanje pre pojave simptoma/povrede/bolesti, odnosno na stanje pre operacije?**
93. Veoma nezadovoljan/na
94. Umereno nezadovoljan/na
95. Diskretno nezadovoljan/na
96. Diskretno zadovoljan/na
97. Umereno zadovoljan/na
98. Veoma zadovoljan/na
99. **Da li ste zadovoljni vašim trenutnim socijalnim životom u odnosu na stanje pre pojave simptoma/povrede/bolesti, odnosno na stanje pre operacije?**
100. Veoma nezadovoljan/na
101. Umereno nezadovoljan/na
102. Diskretno nezadovoljan/na
103. Diskretno zadovoljan/na
104. Umereno zadovoljan/na
105. Veoma zadovoljan/na
106. **Da li ste zadovoljni vašim trenutnim profesionalnim životom u odnosu na stanje pre pre pojave simptoma/povrede/poremećaja/bolesti, odnosno na stanje pre operacije?**
107. Veoma nezadovoljan/na
108. Umereno nezadovoljan/na
109. Diskretno nezadovoljan/na
110. Diskretno zadovoljan/na
111. Umereno zadovoljan/na
112. Veoma zadovoljan/na
